# One‐year outcomes for congenital diaphragmatic hernia

**DOI:** 10.1002/bjs5.50169

**Published:** 2019-05-09

**Authors:** N. Lansdale, D. Wilkinson

**Affiliations:** ^1^ Royal Manchester Children's Hospital Oxford Road, Manchester, M13 9WL UK

We commend the detailed analyses presented by Wang *et al.*, and support the use of routinely collected data such as those from Hospital Episode Statistics (HES) for interrogating surgical outcomes. Reducing unwarranted variation is a priority for the National Health Service and has been the subject of initiatives such as the Getting It Right First Time (GIRFT) programme in paediatric surgery.

Wang and colleagues' findings strike a similar note to work we have done using HES data in paediatric surgery^1^, ^2^; together they highlight the challenges of detecting statistically and clinically significant differences in outcome when investigating rare conditions. The key for future study has to be the development of robust, composite outcome measures that are both clinically relevant and important to patients and families.

We highlight the limitations of HES data and the need to guard against ‘overinterpretation’: Wang *et al.* used ICD‐10 codes for postnatal pulmonary hypertension (PPHN) and pulmonary hypoplasia to investigate their effects on outcome. It is almost certain that these codes were completed somewhat arbitrarily at discharge, and it could be argued that all babies with congenital diaphragmatic hernia (CDH) have PPHN and pulmonary hypoplasia anyway; these analyses therefore seem unhelpful. Furthermore, a centre's outcomes are likely to be determined largely by how severely affected the babies they are looking after are. This case‐mix severity will be influenced greatly by whether or not a centre has inborn babies (rather than those well enough to be transferred in), patterns of termination of pregnancy, and whether or not babies are transferred for specialist care, for instance for extracorporeal membrane oxygenation. HES data lack the granularity required to interrogate these factors accurately. Care should therefore be exercised when interpreting these findings and the reported intercentre variability.
